# Rice aleurone layer specific OsNF-YB1 regulates grain filling and endosperm development by interacting with an ERF transcription factor

**DOI:** 10.1093/jxb/erw409

**Published:** 2016-11-01

**Authors:** Jing-Jing Xu, Xiao-Fan Zhang, Hong-Wei Xue

**Affiliations:** National Key Laboratory of Plant Molecular Genetics, CAS Center for Excellence in Molecular Plant Sciences, Institute of Plant Physiology and Ecology, Shanghai Institutes for Biological Sciences, Chinese Academy of Sciences, 300 Fenglin Road, 200032 Shanghai, China

**Keywords:** Aleurone layer, endosperm development, ERF, GCC box, grain filling, OsNF-YB1.

## Abstract

Grain yield and quality of rice mainly depend on grain filling and endosperm development. Here we report that a rice NUCLEAR FACTOR Y (NF-Y) transcription factor, OsNF-YB1, is specifically expressed in the aleurone layer of developing endosperm and regulates grain filling and endosperm development. Knockdown of *OsNF-YB1* expression by RNAi significantly retarded grain filling, leading to small grains with chalky endosperm as well as altered starch quality. Whereas OsNF-YB1 shows subcellular localization in both the cytosol and the nucleus in roots, it was specifically targeted to the nucleus of aleurone layer cells, which was facilitated by interacting with OsNF-YC proteins preferentially expressed in the aleurone layer. RNA sequencing analysis revealed that genes related to membrane transport and ATP biosynthesis were enriched in the down-regulated category in *OsNF-YB1* RNAi plants, which is consistent with the crucial role of OsNF-YB1 in rice grain filling and endosperm development. Identification of the genome-wide targets of OsNF-YB1 by ChIP sequencing showed that OsNF-YB1 directly regulates genes involved in the transport of nutrients such as sugar and amino acids. Interestingly, different from the binding sites reported for other NF-Y complexes, the GCC box, the binding motif of ERF transcription factors, was enriched in the binding peaks of OsNF-YB1. Indeed, further analyses confirmed the interaction of OsERF#115 with OsNF-YB1, and OsERF#115 directly binds to the GCC box. It is proposed that OsNF-YB1 specifically regulate the transcription of downstream genes during rice endosperm development by forming protein complexes consisting of OsNF-YB1, OsNF-YC and ERF, providing informative insights into the molecular functional mechanisms of the NF-Y factor.

## Highlight

Rice transcription factor OsNF-YB1 is specifically expressed in the aleurone layer of developing endosperm and forms protein complexes consisting of OsNF-YB1, OsNF-YC and ERF to regulate grain filling and endosperm development.

## Introduction

The formation of the embryo and endosperm are key processes in seed development. Endosperm development plays an important and active role in regulating embryo and seed development and the cereal endosperm is important biologically as well as nutritionally and economically as a major source of human food. Elucidation of the molecular mechanisms regulating endosperm development will help to improve grain yield and quality.

Rice endosperm consists of starchy endosperm and the aleurone layer. During grain filling, nutrients are transported into the developing caryopsis through the dorsal vascular bundle, then into the nucellar projection and finally to the endosperm. As there are no plasmodesmatal connections between the maternal and filial tissues (Krishnan and Dayanandan, 2003), the aleurone layer, based on its position and structure, is proposed to play a crucial role in the apoplastic uptake of nutrients into the endosperm.

Due to technical difficulties and the nature of a complex quantitative trait, only a few genes have been identified as participating in the regulation of rice grain filling by genetic or functional genomic studies. *GRAIN INCOMPLETE FILLING 1* (*GIF1*) is expressed in the ovular vascular trace and encodes a cell-wall invertase required for carbon partitioning and grain filling (Wang *et al.*, 2008). The transcription factor *MADS29*, which is preferentially expressed in the nucellar projection and nucellus, regulates the degradation of maternal tissues and hence grain filling (Yin and Xue, 2012). Although studies have revealed the importance of the maternal tissues, little is known about the function in grain filling of the aleurone layer, which is suggested to be crucial for the unloading of nutrients from maternal tissues into filial tissues.

Transcriptional regulation is pivotal in controlling gene expression. Transcription initiation in eukaryotes is mediated by the recruitment of transcription factors (TFs) to the *cis*-elements. Among the first identified *trans*-acting factors and *cis*-elements, NUCLEAR FACTOR Y (NF-Y), also known as CCAAT-binding factor (CBF) and heme-associated protein (HAP), binds to the CCAAT box. NF-Y is composed of NF-YA, NF-YB, and NF-YC subunits in most species, and is evolutionarily conserved in all eukaryotes. Extensive studies of the structure and function of the NF-Y complex in mammals showed that NF-YB and NF-YC form a tight dimer via histone-fold domains, and that this interacts with DNA non-specifically, while NF-YA is responsible for sequence-specific contact with the CCAAT box (Nardini *et al.*, 2013). Interestingly, most eukaryotic genomes have only one or two genes encoding each NF-Y subunit, whereas each subunit is encoded by a family in plants (Petroni *et al.*, 2012; Laloum *et al.*, 2013). The expansion of NF-Y families in plants indicates the possibility of the presence of various NF-Y complexes, allowing for both functional redundancy and functional novelty.

Studies have shown that NF-Y is required for the early embryonic development of mouse, which is consistent with its role in cell-cycle regulation in proliferating cells (Bhattacharya *et al.*, 2003; Oldfield *et al.*, 2014). NF-Y is a key regulator controlling endoplasmic reticulum (ER) organization in mature neurons (Yamanaka *et al.*, 2014) and hepatocytes (Luo *et al.*, 2011). In higher plants, studies have shown that individual subunits of NF-Y play important roles in diverse biological processes including embryogenesis (Lotan *et al.*, 1998), flowering time control (Ben-Naim *et al.*, 2006; Wenkel *et al.*, 2006), ER stress response (Liu and Howell, 2010), drought resistance (Nelson *et al.*, 2007), nitrogen-fixing nodule development (Combier *et al.*, 2006), and endosperm development (Sun *et al.*, 2014; Bai *et al.*, 2015); however, how NF-Y members integratively regulate these processes is poorly understood.

Other transcription factors can interact with NF-Y subunits to regulate the expression of downstream target genes. NF-Y proteins physically interact with bZIP, MADS, and the conserved CCT (for CONSTANS, CONSTANS-LIKE, TOC1) domain proteins to regulate various processes (Masiero *et al.*, 2002; Ben-Naim *et al.*, 2006; Wenkel *et al.*, 2006; Liu and Howell, 2010; Kumimoto *et al.*, 2013).

Our previous studies showed that several members of NF-Y families in rice are co-expressed with endosperm-preferential starch synthesis genes (Fu and Xue, 2010). Further systemic studies on the functions and molecular mechanism of NF-Y members in developing endosperm and grain filling showed that rice OsNF-YB1, which is specifically expressed in the aleurone layer during early stages of endosperm development, plays an important role in regulating grain filling by forming a transcriptional complex with NF-YC and an ERF transcription factor. These results provide direct evidence of aleurone layer function in endosperm development and provide clues to the molecular mechanisms of the NF-Y family.

## Materials and methods

### Plant materials and growth conditions

Japonica rice (*Oryza sativa* cv Zhonghua11, ZH11) was used for rice transformation. Transgenic rice plants were grown in a phytotron with a 12 h light (28 ^o^C)–12 h dark (22 ^o^C) cycle. To measure grain-related traits, plants were grown in an experimental field under natural conditions.

To generate *OsNF-YB1* (LOC_Os02g49410) RNAi plants, a 311-bp fragment specific to the *OsNF-YB1* coding region (15–325 nt) was amplified by PCR and inserted in the sense orientation into the *Hin*dIII/*Pst*I sites of the pSK-Int vector. The same fragment was subsequently cloned and inserted in the antisense orientation into the *Kpn*I/*Bam*HI sites of pSK-Int carrying the sense fragment. Finally, the fragment in pSK-Int containing the 478-bp intron flanked with two 311-bp opposite fragments was digested with *Hin*dIII and cloned into pUN1301, resulting in the RNAi construct *OsNF-YB1*. The resultant construct was transformed into ZH11 by *Agrobacterium*-mediated transformation and homozygous T2 lines were analyzed. Primers for creating the RNAi construct are listed in the Supplementary Table S1 at *JXB* online.

To generate the *OsNF-YB1*-overexpressing plants, an approximately 7.3-kb DNA fragment containing the whole *OsNF-YB1* gene and ~5.6-kb upstream of the translation initiation codon was digested with *Xba*I/*Sac*I from bacterial artificial chromosome clone OSJNBa0096K12 (Arizona Genomics Institute) and subcloned into the *Xba*I/*Sac*I sites of the pCAMBIA1300 vector (CAMBIA) for transformation. Homozygous T2 rice lines were analyzed.

### Quantitative RT-PCR analysis

Total RNA was extracted using TRIzol reagent (Life Technologies) and reverse transcribed into cDNA according to the manufacturer’s instructions (ReverTra Ace qPCR RT Master Mix with gDNA Remover; Toyobo). Quantitative RT-PCR (qRT-PCR) was performed by using the SYBR Green Realtime PCR Master Mix (Toyobo). Rice *Actin* (LOC_Os03g50885) gene was used as an internal standard to normalize the expression of tested genes. Relevant primer sequences are listed in Supplementary Table S1.

### Promoter–reporter fusion studies

The ~3.4-kb putative promoter region of *OsNF-YB1* (upstream of ATG) was amplified by PCR and subcloned into *Xba*I/*Bam*HI sites of pCAMBIA1300+pBI101. The resultant construct was transformed into ZH11 and independent lines of positive T2 transgenic progeny were used to detect GUS activity. Photography was performed using a Nikon microscope (SMZ1500) with a digital camera. Primer sequences are listed in Supplementary Table S1.

### *In situ* hybridization analysis

A 385-bp specific fragment of *OsNF-YB1* was amplified by PCR (primer sequences are listed in Supplementary Table S1), subcloned into pGEM-T easy vector (Promega), and used as a template to generate digoxigenin-labeled sense and antisense probes (Roche). Caryopses of ZH11 were fixed by 4% paraformaldehyde in 0.1 M sodium phosphate buffer, dehydrated through a graded ethanol series, replaced with xylene, embedded in paraffin (Sigma-Aldrich), and sectioned at 8 mm. *In situ* hybridization was performed according to the previous description (Luo *et al.*, 1996).

### Measurement of grain quality

Grain weight, apparent amylose content (AAC), and the chain length distributions of amylopectin were analyzed as described previously (Fu and Xue, 2010). Rapid Visco Analyzer (RVA) profiles were obtained with an RVA-4 series model (Newport Scientific, Warriewood, Australia). At least 1000 milled grains were used for measuring percentage of grains with chalkiness (PGWC) and degree of endosperm chalkiness (DEC) on a chalkiness visualizer.

### Yeast two-hybrid analysis

Whole *OsNF-YB1* coding region was amplified by PCR and subcloned into pGBKT7 bait plasmid. Coding regions of *OsNF-YC8* (LOC_Os01g01290), *OsNF-YC9* (LOC_Os01g24460), *OsNF-YC10* (LOC_Os01g39850), *OsNF-YC11* (LOC_Os05g23910), *OsNF-YC12* (LOC_Os10g11580), *OsERF#074* (LOC_Os05g41780), *OsERF#114* (LOC_Os06g42910), *OsERF#115* (LOC_Os08g41030), and *OsERF#072* (LOC_Os09g26420) were amplified and cloned into pGADT7 prey plasmid. Yeast strain AH109 was co-transformed with specific bait and prey constructs through a lithium acetate-mediated method. Interactions were tested using SD/–Leu/–Trp/–His/–Ade medium. Primer sequences are listed in Supplementary Table S1.

### Yeast one-hybrid analysis

A twenty base pair DNA fragment of *Os07g19070* promoter containing the intact GCC box (or mutated GCC box, primer sequences are listed in Supplementary Table S1) was repeated three times and inserted into pHIS2.1 (Clontech). *OsERF#115* or *NF-YB1* was fused to GAL4 transcriptional activation domain (AD). Yeast strain AH109 was co-transformed with the indicated constructs through a lithium acetate-mediated method and a one-hybrid assay was performed following the manufacturer’s manual (Clontech).

### Transcriptional activity assay in yeast cells

Coding regions of *OsERF#115* were amplified by PCR, subcloned into pGBKT7 and fused with the BD domain. The resultant construct was transformed into AH109 and transcriptional activity was examined by observing the yeast cell growth on SD/–Trp/–His/–Ade medium.

The coding region of *OsERF#115* was amplified by PCR, subcloned into pBridge and fused with the BD domain. The *OsNF-YB1* coding region was then subcloned into the pBridge-OsERF#115 construct. Coding regions of *OsNF-YC11* or *OsNF-YC12* were subcloned into modified pGADT7 without GAL4-activation domain. Yeast strain AH109 was co-transformed with the indicated constructs and transformed yeast cells were selected on SD/–Leu/–Trp medium. Transcriptional activity was examined by observing the cell growth on SD/–Leu/–Trp/–His/–Met medium.

Sequences of primers used are listed in Supplementary Table S1.

### Subcellular localization studies

Coding regions of *OsNF-YB1* were amplified by PCR and subcloned into pA7 vector. The resultant construct was introduced into onion epidermal cells (pA7 vector as control) and green fluorescence was observed with a confocal laser scanning microscope (Olympus FV1000).

For transient expression of fusion protein in *Nicotiana benthamiana* (tobacco) leaf epidermal cells, a DNA fragment in pA7 containing *OsNF-YB1*-*GFP* was digested with *Hin*dIII/*Sac*I and cloned into PHB vector (Mao *et al.*, 2005). Coding regions of *OsNF-YC2* (LOC_Os03g14669), *OsNF-YC8*, *OsNF-YC9*, *OsNF-YC10*, *OsNF-YC11* and *OsNF-YC12* were amplified by PCR and subcloned into a modified pCambia1300 containing mCherry reporter. Constructs were co-transformed with the P19 silencing suppressor into tobacco leaves through *Agrobacterium* infiltration and examined with a confocal laser scanning microscope (Olympus FV1000) after infiltration for 2 days.

To construct *pUbi:OsNF-YB1-GFP*, a DNA fragment in pA7 containing *OsNF-YB1-GFP* was digested with *Xho*I/*Eco*RI and cloned into pUN1301. The resultant construct was transformed into ZH11 and homozygous T2 lines were analyzed.

All primer sequences are listed in the Supplementary Table S1.

### Preparation of rice aleurone layer cells

The starchy endosperm and embryo of caryopses were removed, and aleurone layer cells were isolated from the remaining caryopsis coats as described previously (Hay and Spanswick, 2007). Inner layers separated mechanically from the caryopsis coats were treated with incubation medium (IM) containing 0.1% (w/v) pectolyase Y-23 for 1.5 h; then the coats were transferred back to an equal volume of IM and manually sheared with a fingertip to separate aleurone layer cells from the maternal tissues. Maternal tissues were removed and aleurone layer cells were collected after centrifugation.

### Co-immunoprecipitation assay

For co-immunoprecipitation (Co-IP) analysis, constructs *OsNF-YB1-GFP*, *OsNF-YC11-mCherry*, or *OsNF-YC12-mCherry* were firstly co-transformed with the P19 silencing suppressor into tobacco leaves. After 2 days, the infiltrated leaves were ground in liquid nitrogen, lysed with the extraction buffer (50 mM Tris–HCl, pH 7.5, 1 mM EDTA, 150 mM NaCl, 10% glycerol, 1% NP-40, and Roche Complete Protease Inhibitor Cocktail), and centrifuged at ~10 000 *g* for 20 min. Supernatant was incubated with anti-GFP antibody (ab290; Abcam) and coupled to Dynabeads Protein G (Life Technologies) for 2 h. Beads were washed three times with wash buffer (100 mM Tris–HCl, pH 7.5, 1 mM EDTA, 10% glycerol, 75 mM NaCl, 0.1% Triton X-100). The bound proteins were eluted with 2× SDS sample buffer, and subjected to immunoblot analysis using monoclonal anti-GFP antibody (sc-9996; Santa) and anti-mCherry antibody (ab125096; Abcam).

### RNA-seq and GO term analysis

One hundred caryopses at 8 days after flowering (DAF) of 20 independent plants were used to isolate the aleurone layer cells as described above. Throughout the process, cordycepin (3′-deoxyadenosine; Sigma) was added to a final concentration of 1 mM to inhibit transcription. Total RNA was extracted using Trizol, and Illumina sequencing libraries were constructed according to the manufacturer’s instructions. The libraries were sequenced with Hiseq2500. Reads were aligned to the rice genome (MSU7.0) using Tophat. Differentially expressed genes were indicated as false discovery rate (FDR)<0.05 with Cuffdiff.

Gene ontology (GO) enrichment was performed using BiNGO. GO information from the gene ontology website (http://www.geneontology.org/), Rice Genome Annotation (http://rice.plantbiology.msu.edu/), and *Arabidopsis* annotation (ftp://ftp.arabidopsis.org/home/tair/Ontologies/Gene_Ontology/) was used. The hypergeometric test was used with subsequent Benjamini and Hochberg FDR corrections. GO annotation terms were considered significant if the corrected *P*-value was <0.05 and if there were at least five genes associated with the same annotation.

### ChIP assay and ChIP-seq analysis

Two independent transgenic lines expressing *pUbi:OsNF-YB1-GFP* (lines 16 and 22) were used for ChIP assay. For each biological repeat, 1 g of caryopsis coats at 8–12 DAF was harvested. Tissue fixation, nuclei extraction, and chromatin immunoprecipitation using anti-GFP antibody (ab290; Abcam) were performed as described previously (Bowler *et al.*, 2004). Normal rabbit IgG (10500C; Invitrogen) was used as a negative control. Immunoprecipitated and input DNA were purified with a PCR purification kit (Qiagen) and stored at −70°C for analysis.

Immunoprecipitated and input DNA, pooled from four independent ChIP experiments, were end-repaired and supplemented with A base followed by ligation with adapters. The ChIP-seq DNA libraries were amplified by PCR for 15 cycles and sequenced with Hiseq2500.

The short reads given were aligned to the rice genome (MSU7.0) by Bowtie2 with default parameters. MACS2 (Zhang *et al.*, 2008) was used for calling stronger but narrower peaks (*q*-value<0.05) and weaker but broader regions (*q*-value<0.1) with default settings. Overlapping peaks (having a stronger but narrower peak in at least one replicate) between two biological replicates were used for further analysis. Target genes were defined when overlapping peaks appeared on their genic or within 3-kb upstream regions. The summits of overlapping peaks were used to define the location types in the genome as described previously (Lu *et al.*, 2013). Motif enrichment analysis was performed by using DREME (Bailey, 2011) with default parameters. Sequences of 100 bp around the summits of overlapping peaks that appeared within 3-kb upstream regions were filtered by RepeatMasker (http://repeatmasker.org), and then used for motif search.

For ChIP-seq validation, the prepared DNA in ChIP was applied for qPCR analysis. All primer sequences are listed in Supplementary Table S1.

ChIP-seq and RNA-seq data generated by this study have been deposited in the Gene Expression Omnibus database (GSE72187).

## Results

### *OsNF-YB1* is specifically expressed in aleurone layer of rice developing endosperm

A member of the *NF-Y* families, *OsNF-YB1*, is positively correlated with the endosperm-preferentially expressed starch synthesis genes by co-expression analysis (Fu and Xue, 2010). To study the physiological role of OsNF-YB1 in endosperm development, the expression pattern of *OsNF-YB1* in various tissues was firstly analyzed by quantitative RT-PCR (qRT-PCR). Results showed that *OsNF-YB1* was specifically expressed in the developing caryopses and reached a maximum at 8 days after fertilization (DAF; Supplementary Fig. S1A). Further promoter-β-glucuronidase (*GUS*) fusion analysis revealed that *OsNF-YB1* was expressed in the outer surface of the endosperm, possibly the aleurone layer of developing caryopses (see Supplementary Fig. S1B), which is similar to a report by Bai *et al.* (2015).

**Fig. 1.**
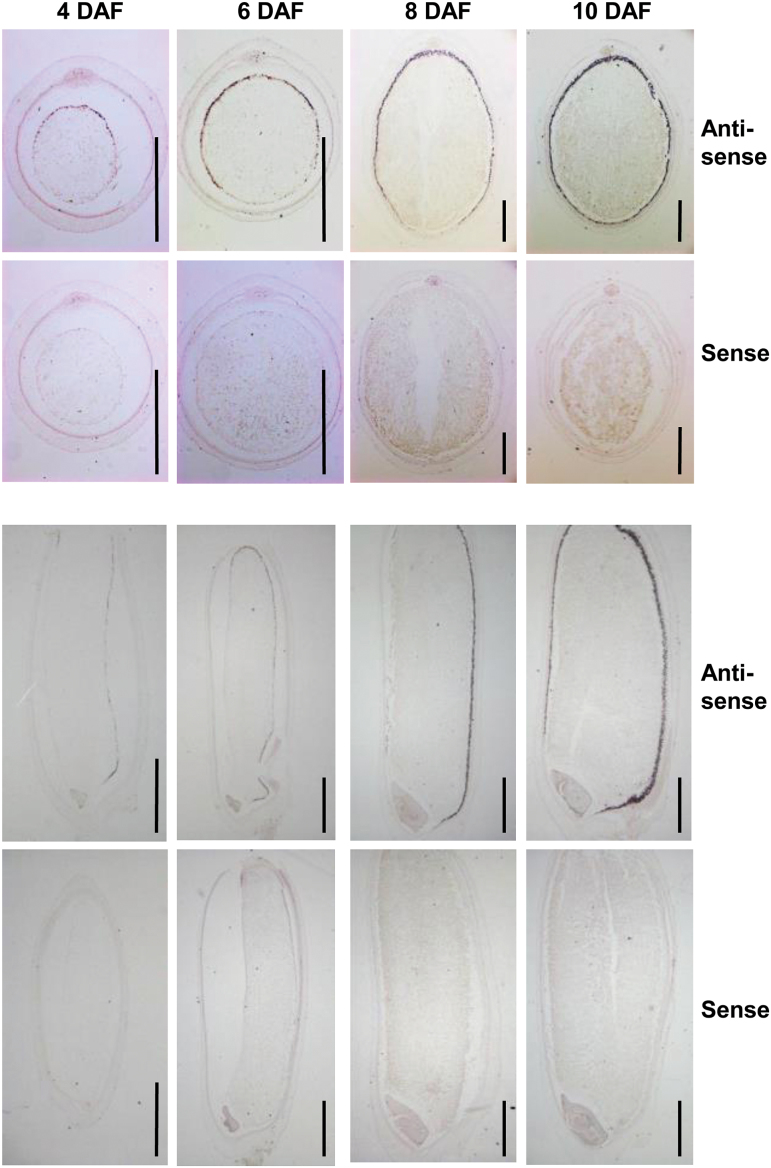
*OsNF-YB1* is specifically expressed in the aleurone layer of rice endosperm. RNA *in situ* hybridization analysis of the *OsNF-YB1* transcripts during seed development. Transverse (upper, bars: 500 µm) and longitudinal (bottom, bars: 1 mm) sections of seeds at 4, 6, 8, and 10 DAF were analyzed. Sense probe was used as control.

Rice endosperm development includes four stages: coenocytic, cellularization, differentiation, and maturation. After completion of cellularization, differentiation of the aleurone cells starts in the periphery of the endosperm adjacent to the dorsal vascular bundle and spreads laterally to the ventral side. The aleurone layer is composed of several cell layers on the dorsal side and one to two layers on the lateral and ventral sides. To determine more precisely the spatial and temporal expression patterns of *OsNF-YB1*, RNA *in situ* hybridization analysis was performed using caryopses at different developmental stages. Results showed that *OsNF-YB1* was expressed in the dorsal aleurone layer cells at 4 DAF, immediately after the completion of cellularization. Along with endosperm differentiation, *OsNF-YB1* was expressed at the dorsal and lateral aleurone layer cells, and in the entire aleurone layer at 10 DAF (Fig. 1). These results indicate the specific expression *OsNF-YB1* in the aleurone layer and its possible role in developing endosperm.

### Suppressed expression of *OsNF-YB1* results in the reduced grain filling

To study the physiological role of *OsNF-YB1*, an RNA interference (RNAi) construct containing a non-conserved region of *OsNF-YB1* was generated and transformed into rice (Zhonghua11, ZH11). Analysis of the T0 transgenic lines (lines 2, 4, 8, and 12) revealed they were independent lines by genomic Southern blot (see Supplementary Fig. S2A). Further analysis confirmed the significantly suppressed *OsNF-YB1* expression in the T2 generation of transgenic lines, while the expression levels of *OsNF-YB6* and *OsNF-YB8*, which present nucleotide sequences homologous with *OsNF-YB1*, were not affected (Supplementary Fig. S2B).

**Fig. 2.**
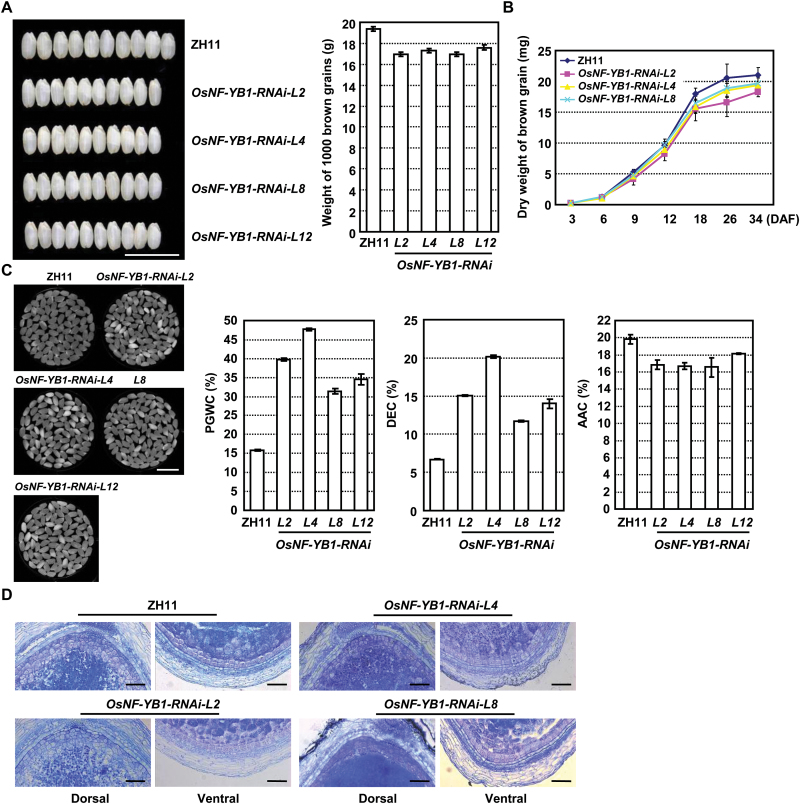
Suppressed expression of *OsNF-YB1* results in reduced grain filling. (A) Observation and calculation showed that brown grains of *OsNF-YB1* RNAi plants were smaller compared with ZH11 (left, bar: 1 cm) and 1000-brown-grain weight was reduced (right). Data are shown as mean±SD from ten replicates. (B) Analysis of the time course of seed dry weight revealed reduced grain filling of *OsNF-YB1* RNAi plants. Data are shown as mean±SE (*n*=10). (C) Observation and measurement showed that endosperm of *OsNF-YB1* RNAi plants displayed higher chalkiness including higher percentage of grain with chalkiness (PGWC) and degree of endosperm chalkiness (DEC), and lower apparent amylose content (AAC). Data are shown as mean±SD from three replicates. Bar: 1 cm. (D) Observation of transverse sections of seeds revealed a normal aleurone layer of *OsNF-YB1* RNAi plants. Dorsal (left) and ventral side (right) of seeds at 10 DAF are shown. Bars: 200 µm.

Phenotypic observations showed that compared with ZH11, there were no obvious growth differences of *OsNF-YB1* RNAi plants, including height, flowering timing, panicle number per plant, and grain number per panicle (see Supplementary Fig. S2C–E), while the brown grains were smaller, resulting in a reduced 1000-brown-grain weight (Fig. 2A). Detailed measurement showed that smaller brown grain was mainly due to reduced grain width and thickness, and grain length was not altered (Supplementary Fig. S2F). Further comparison revealed the decreased grain-filling rate of *OsNF-YB1* RNAi plants during grain development (between 6 and 12 DAF; Fig. 2B).

Interestingly, compared with those of ZH11, *OsNF-YB1* RNAi grains displayed higher chalkiness, including a higher percentage of grain with chalkiness (PGWC) and degree of endosperm chalkiness (DEC) (Fig. 2C). The apparent amylose content (AAC) of *OsNF-YB1* RNAi grain endosperm was reduced (Fig. 2C), and analysis of the structural changes of amylopectin showed that the proportion of chains with degree of polymerization (DP) in the range of 6–7 was increased, whereas the proportion of chains with DP in the range of 8–17 was significantly decreased (see Supplementary Fig. S3A). The Rapid Visco Analyzer (RVA) profile provides a comprehensive evaluation of starch quality and further analyses showed that *OsNF-YB1* RNAi grains had a different RVA profile (Supplementary Fig. S3B), indicating the obviously altered physicochemical characteristics of starch under suppressed *OsNF-YB1*.

In maize and barley, a number of mutants with disorganized aleurone layers produce shrunken grains. In contrast to these mutants, the *OsNF-YB1* RNAi seeds presented a normal aleurone layer composed of multilayers at the dorsal side adjacent to the major vascular bundle and one or two layers at the ventral and lateral sides (Fig. 2D), suggesting that OsNF-YB1 is not involved in the regulation of aleurone cell organization and abnormal *OsNF-YB1* RNAi grains may be caused by reduced nutrient transport through aleurone layer cells.

The entire *OsNF-YB1* gene driven by its own promoter was transformed into rice (see Supplementary Fig. S4) and analysis of seven selected independent *OsNF-YB1*-overexpressing lines showed that compared with ZH11, the PGWC and DEC of five *OsNF-YB1*-overexpressing lines were reduced (Table 1), suggesting that increased *OsNF-YB1* expression may improve grain quality by reducing grain chalkiness, and suggesting the possible application of *OsNF-YB1* in rice breeding.

**Table 1.** *Grain-related traits of ZH11 and* OsNF-YB1*-overexpressing lines* The data are shown as mean±SD from three replicates. Analysis by two-tailed unpaired *t* tests indicates significant differences in 1000-brown-grain weight, percentage of grain with chalkiness (PGWC) and degree of endosperm chalkiness (DEC) between ZH11 and *OsNF-YB1*-overexpressing lines (**P<0.01).

Line	1000-brown-grain weight (g)	PGWC (%)	DEC (%)
ZH11	19.18 ± 0.09	19.40 ± 0.26	8.26 ± 0.36
*pOsNF-YB1:OsNF-YB1*			
L1	19.76 ± 0.17	16.14 ± 0.44**	5.73 ± 0.11**
L3	19.34 ± 0.23	13.00 ± 0.89**	5.15 ± 0.19**
L5	20.08 ± 0.09**	12.44 ± 0.46**	4.28 ± 0.17**
L6	17.29 ± 0.26**	18.73 ± 0.58	7.18 ± 0.04
L7	19.54 ± 0.06**	12.00 ± 0.02**	4.63 ± 0.18**
L8	16.60 ± 0.12**	19.74 ± 0.21	8.70 ± 0.01
L14	20.47 ± 0.10**	9.75 ± 1.24**	3.46 ± 0.30**

In addition, observation showed that compared with ZH11, OsNF-YB1 RNAi or *OsNF-YB1*-overexpressing plants displayed no differences in seed germination ratio and viability.

### OsNF-YB1 is specifically localized in the nucleus of aleurone cell through interacting with OsNF-YC11 or OsNF-YC12

**Fig. 3.**
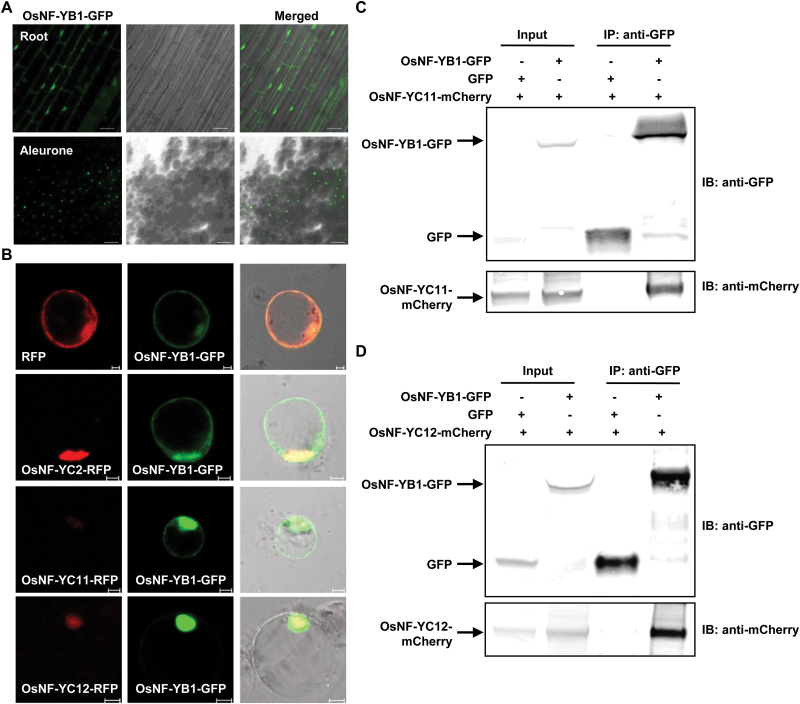
OsNF-YC11 and OsNF-YC12 interact with OsNF-YB1 *in vivo* and mediate the nuclear localization of OsNF-YB1. (A) Observation of OsNF-YB1 subcellular localization showed a dual cytosolic–nuclear localization in root cells and nucleus-specific localization in aleurone layer cells. Root of young seedlings and aleurone layer cells of 10 DAF seeds expressing *pUbi:OsNF-YB1-GFP* were observed. Bars: 50 µm (upper) or 20 µm (bottom). (B) OsNF-YB1-GFP protein showed a dual cytosolic–nuclear localization in the presence of red fluorescent protein (RFP) or OsNF-YC2-RFP, and was translocated to nucleus of rice protoplast cells in the presence of OsNF-YC11-RFP or OsNF-YC12-RFP. Bars: 5 µm. (C, D) Co-immunoprecipitation analysis revealed the interaction of OsNF-YB1-GFP and OsNF-YC11-mCherry (C), and OsNF-YB1-GFP and OsNF-YC12-mCherry (D) in tobacco cells. Total protein extracts (Input) or immunoprecipitated (IP) fractions using an anti-GFP antibody were analyzed using anti-GFP or anti-mCherry antibodies.

To investigate the subcellular localization of OsNF-YB1 protein, a transient expression assays using onion epidermal cells or tobacco (*Nicotiana benthamiana*) cells showed that the green fluorescent protein (GFP)-tagged OsNF-YB1 protein was located in both the cytosol and the nucleus (see Supplementary Fig. S5), which was the same as with OsNF-YB1 in rice root cells expressing *pUbi:OsNF-YB1-GFP* (Fig. 3A). However, different from the location in root cells, observation of the isolated aleurone layer cells of developing caryopses showed that OsNF-YB1-GFP protein was located in the nucleus only (Fig. 3A), which is consistent with the role of OsNF-YB1 in transcriptional regulation.

Studies in fungi and mammals showed that the nuclear import of NF-YB and NF-YC requires heterodimer formation (Steidl *et al.*, 2004; Kahle *et al.*, 2005), which is largely unknown in plants. Nuclear localization of OsNF-YB1 suggests that there are possible unknown NF-YC subunits that may interact with OsNF-YB1 and mediate the OsNF-YB1 nuclear localization in aleurone layer cells. Up to now, seven *NF-YC* genes in rice genome have been annotated (named as *OsNF-YC1* to *OsNF-YC7*; Petroni *et al.*, 2012) and nine new *OsNF-YC* genes were identified from a data set of predicted *Oryza sativa* L. ssp. *japonica NF-YC* genes retrieved from the PlantTFDB (Jin *et al.*, 2014). Our previous studies (Xue *et al.*, 2012) showed that five *NF-YC* genes (Os01g01290, Os01g24460, Os01g39850, Os05g23910 and Os10g11580, which were named as *OsNF-YC8* to *OsNF-YC12*) are preferentially expressed in endosperm (see Supplementary Fig. S6A), and thus the possible interactions of them with OsNF-YB1 was examined by yeast two-hybrid analysis. Results showed that all these five NF-YCs interact with OsNF-YB1 in yeast cells (Supplementary Fig. S6B). A further transient expression assay using tobacco epidermal cells showed that OsNF-YC11-mCherry and OsNF-YC12-mCherry co-expression mediated the OsNF-YB1-GFP nuclear localization (strong GFP signals were only observed in nucleus; Supplementary Fig. S6C), while OsNF-YB1-GFP was observed in both the cytosol and nucleus when co-expressing with mCherry, OsNF-YC8-mCherry, OsNF-YC9-mCherry, OsNF-YC10-mCherry, or OsNF-YC2-mCherry (*OsNF-YC2* was preferentially expressed in the vegetative tissues by microarray analysis, Supplementary Fig. S6A).

Consistent with this, qRT-PCR analysis revealed that compared with *OsNF-YC8*, *OsNF-YC9* and *OsNF-YC10*, both *OsNF-YC11* and *OsNF-YC12* were significantly highly expressed in aleurone layer (see Supplementary Fig. S6D). Further transient expression assay using rice protoplast cells showed that OsNF-YC11 or OsNF-YC12 mediated the OsNF-YB1 nuclear localization in rice cells (Fig. 3B), and co-immunoprecipitation assay using tobacco revealed the *in vivo* interaction of OsNF-YB1 with OsNF-YC11 or OsNF-YC12 (Fig. 3C, D), confirming that aleurone layer-preferentially expressed NF-Y subunits OsNF-YC11 and OsNF-YC12 mediated the OsNF-YB1 nuclear localization through interactions.

### *OsNF-YB1* regulates endosperm development by modulating multiple genes/processes

**Fig. 4.**
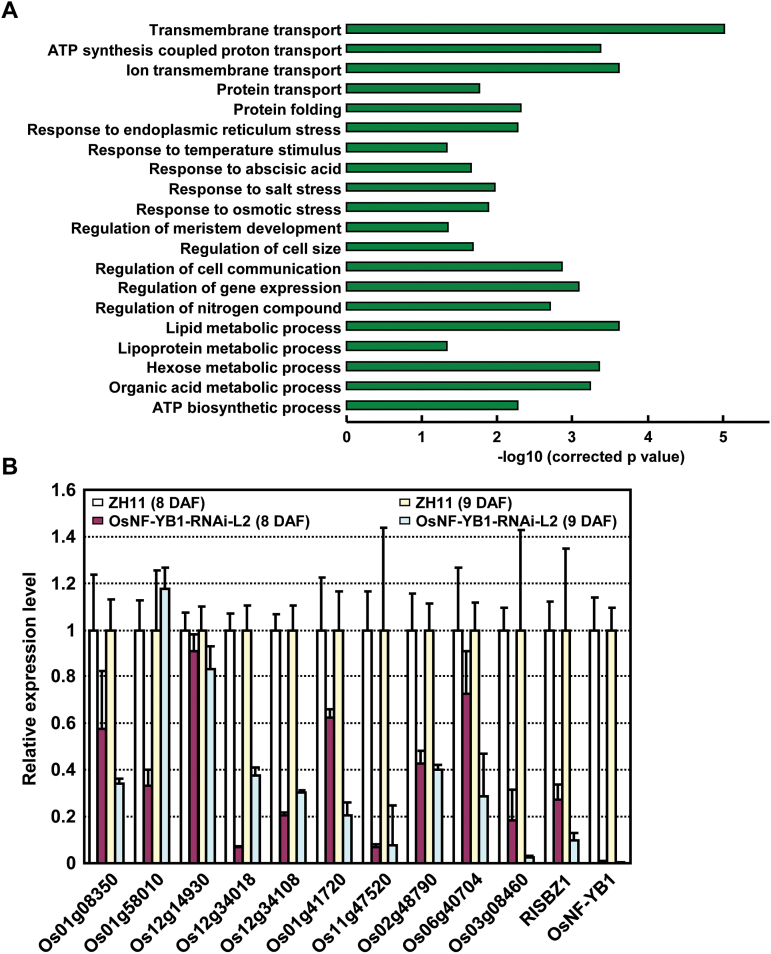
Genome-wide analyses of the OsNF-YB1-regulated network. (A) Selected enriched GO terms of the 960 down-regulated genes by RNA-seq using aleurone layer cells at 8 DAF of *OsNF-YB1* RNAi plants. Hypergeometric test was used with subsequent Benjamini and Hochberg false discovery rate corrections. Only GO terms with a corrected *P*-value <0.05 and at least five annotated genes were kept. Length of bars represents negative logarithm (base 10) of the corrected *P*-value. (B) qRT-PCR analysis confirmed the down-regulation of genes in aleurone layer cells of the *OsNF-YB1* RNAi plants. Relative expression of examined genes (listed in Supplementary Table S2) was calculated (expression of the corresponding gene in ZH11 was set as 1.0) and data are shown as mean±SE (*n*=3).

To study the functional mechanism of OsNF-YB1, differentially expressed genes under suppressed *OsNF-YB1* were identified by RNA sequencing (RNA-seq) using aleurone layer cells of ZH11 and *OsNF-YB1* RNAi plants. Aleurone layer cells were isolated enzymatically from the caryopses at 8 DAF (Hay and Spanswick, 2007) and total RNAs were extracted for sequencing. A total of 1171 genes were characterized showing differential expression (FDR<0.05), and further analysis by gene ontology (GO) annotation showed no significantly over-represented processes of the 211 up-regulated genes, while of the 960 down-regulated genes, transport, ATP synthesis, protein folding, response to stimuli, and metabolic processes were enriched (Fig. 4A), which is consistent with the decreased grain-filling rate (transport and ATP synthesis) and increased chalkiness (protein folding) of *OsNF-YB1* RNAi plants and indicates a crucial role of OsNF-YB1 through transcriptional regulation.

qRT-PCR analyses confirmed the reduced expression levels of genes encoding ATP synthase and transporter under suppressed *OsNF-YB1* expression (Fig. 4B). In addition, previous studies showed that *RISBZ1* is expressed in aleurone and subaleurone layer of developing endosperm (Onodera *et al.*, 2001) and plays an essential role during grain filling (Kawakatsu *et al.*, 2009). Reduced *RISBZ1* expression (Fig. 4B) suggested that *RISBZ1* is regulated by OsNF-YB1 to affect the grain filling.

### Genome-wide determination of OsNF-YB1 binding sites

The study by Bai *et al.* (2015) showed that OsNF-YB1 directly regulates the expression of the sucrose transporter and hence sugar loading to rice endosperm, resulting in altered grain filling. Based on multiple genes or processes being regulated by OsNF-YB1, the mechanism of OsNF-YB1’s effects was further investigated on a genome-wide basis.

**Fig. 5.**
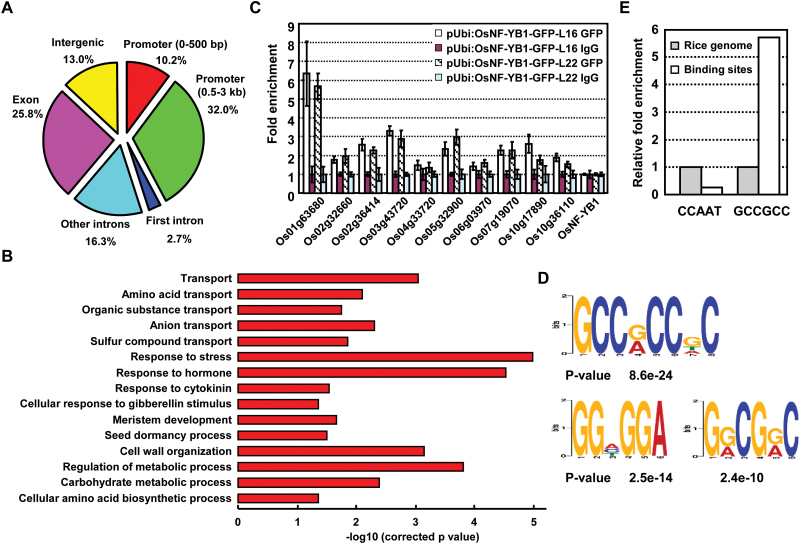
Overview of ChIP-seq data and verification of OsNF-YB1 binding sites. (A) Distribution of the OsNF-YB1 binding peaks relative to gene structure. (B) Selected enriched GO terms of genes associated with OsNF-YB1 binding by ChIP-Seq analysis. Only GO terms with a corrected *P*-value <0.05 and at least five annotated genes were kept. Length of bars represents negative logarithm (base 10) of the corrected *P*-value. (C) ChIP-qPCR validation of the selected OsNF-YB1 targets. Enrichment of the ten candidate OsNF-YB1 binding targets (listed in Supplementary table S2) was examined. *OsNF-YB1*, which has no enrichment in ChIP-seq data, was used as the negative control. Fold enrichment of DNA was calculated as the ratio between αGFP and IgG. Data are presented as means±SD from three replicates. (D) The most enriched motifs identified by DREME in OsNF-YB1 binding peaks that appeared within 3 kb upstream regions of genes. (E) Enrichment of GCC box and CCAAT box compared with the promoter regions (0–3 kb) of reference rice genome. CCAAT box was not enriched in the binding sites.

Target genes of OsNF-YB1 were firstly identified using ChIP sequencing (ChIP-seq). Based on the specific expression of *OsNF-YB1*, caryopsis coats (the tissues that remain after the starchy endosperm and embryo are removed mechanically from the caryopsis) at 8–12 DAF were collected from two independent transgenic lines expressing *pUbi:OsNF-YB1-GFP*, and then used to carry out the ChIP assays with an anti-GFP antibody (see Supplementary Fig. S7). ChIP-seq data were analyzed with the statistical software MACS2 (Zhang *et al.*, 2008) and 933 overlapping peaks of two biological replicates were finally identified, which were considered as high-confidence OsNF-YB1 binding peaks for further analysis. Consistent with the function of OsNF-YB1 as a transcription factor, genome distribution analysis revealed that most of the OsNF-YB1 binding peaks were in the DNA regions 3 kb upstream of the transcription start site (Fig. 5A).

The 933 binding peaks linked to 743 neighbor genes and are considered as the OsNF-YB1 target genes. Functional analysis by GO annotation showed that many terms related to nutrients transport (including amino acids, organic substances, anions) were significantly enriched (Fig. 5B). ChIP-qPCR assays further confirmed the direct binding of OsNF-YB1 to selected genes, including sugar transporters and transcription factors (Fig. 5C), which is consistent with the report by Bai *et al.* (2015).

Interestingly, a search for motifs using DREME (Bailey, 2011) led to the identification of three significantly enriched motifs (Fig. 5D) and analysis using TOMTOM (Gupta *et al.*, 2007) showed that two motifs were similar to the GCC box (GCCGCC), the binding motif of ERF1 protein (Godoy *et al.*, 2011). Of the 933 OsNF-YB1 binding peaks, the GCC box was found in 701 peaks and was enriched in the binding peaks compared with the rice genome (Fig. 5E), while the CCAAT motif was not enriched, which is consistent with the previous studies showing that OsNF-YB1 is impaired in NF-YA association, thus precluding CCAAT binding (Masiero *et al.*, 2002).

### OsNF-YB1 interacts with ERF transcription factor

The motif GCC box is recognized by ERF subfamily proteins belonging to the AP2/ERF superfamily. Since NF-YB–NF-YC dimer binds to DNA non-specifically (Nardini *et al.*, 2013), it is suggested that OsNF-YB1 targets the GCC box through interaction with an ERF member. Eighty-three rice genes are assigned to the ERF subfamily (Nakano *et al.*, 2006) and analysis of gene transcription based on the RNA-seq data and previous microarray data showed that *OsERF#115* had the highest expression in aleurone layer (see Supplementary Table S3), and it was selected for further analysis. In addition we selected and analyzed at same time *OsERF#072* and *OsERF#074*, which are relatively highly expressed in aleurone layer, *OsERF#114*, which is preferentially expressed in developing endosperm but lowly expressed in aleurone layer (Supplementary Table S3, and *OsERF#115*, which is preferentially expressed in developing endosperm.

**Fig. 6.**
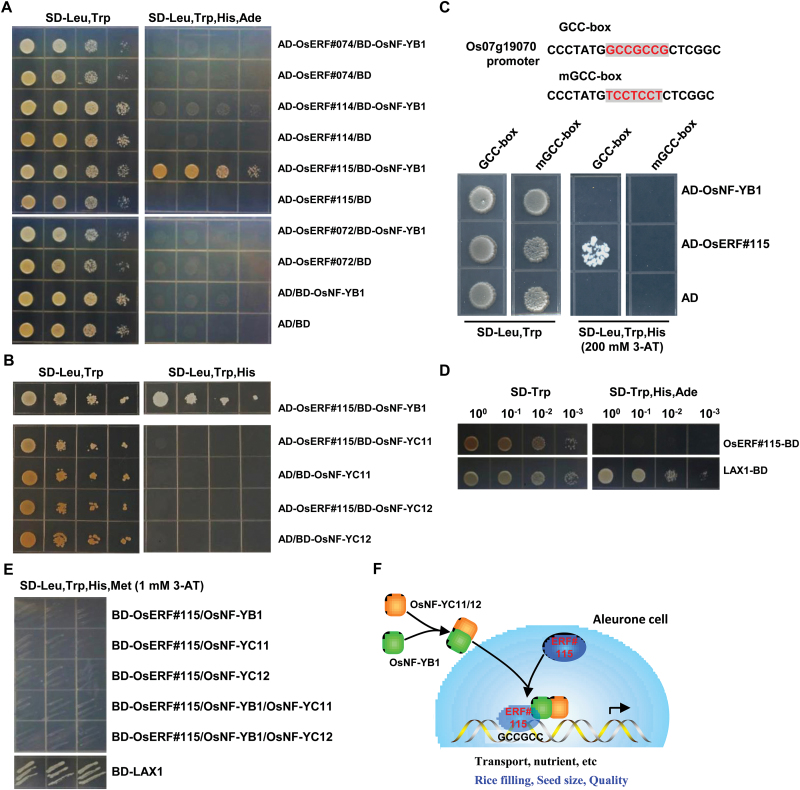
OsNF-YB1 interacts with transcription factor OsERF#115. (A) Yeast two-hybrid assays revealed the interactions between OsNF-YB1 and OsERF#115. Serial dilutions (10×) of yeast cells expressing the indicated proteins were plated onto the non-selective medium (SD/–Leu/–Trp) (left) or selective medium (SD/–Leu/–Trp/–His/–Ade) (right) and cell growth were observed. OsERF members and OsNF-YB1 were fused to activation domain (AD) and binding domain (BD), respectively. (B) Yeast two-hybrid assays showed that there was no interaction between OsERF#115 and OsNF-YC11 or OsNF-YC12. Serial dilutions (10×) of yeast cells expressing the indicated proteins were plated onto the non-selective medium (SD/–Leu/–Trp) (left) or selective medium (SD/–Leu/–Trp/–His) (right) and cell growth was observed. OsERF#115 and OsNF-YC11/OsNF-YC12 were fused to AD and BD, respectively. (C) Yeast one-hybrid assays revealed that OsERF#115 binds to, while OsNF-YB1 does not, the GCC box. OsERF#115 or OsNF-YB1 was fused to GAL4 transcriptional activation domain (AD) and used as prey. DNA fragment of Os07g19070 promoter containing the GCC box (or with mutated bases) was repeated three times and fused to pHIS2 as baits. Yeast cells co-transformed with indicated prey or bait vectors were grown on non-selective medium (SD/–Leu/–Trp) (left) or selective medium (SD/–Leu/–Trp/–His, 200 mM 3-amino-1,2,4-triazole (3-AT)) (right) and cell growth was observed. (D) Analysis showed that OsERF#115, which was fused to the GAL4 binding domain (BD) of pGBKT7, does not present transactivational activity. LAX1-BD was used as positive control. (E) There was no transactivational activity of yeast cells expressing OsERF#115, OsERF#115/OsNF-YB1, OsERF#115/OsNF-YC11, OsERF#115/OsNF-YC12, or OsERF#115/OsNF-YB1/OsNF-YC11, OsERF#115/OsNF-YB1/OsNF-YC12. OsERF#115 was fused to the GAL4 binding domain (BD, pBridge vector). OsNF-YB1 was subcloned into pBridge-OsERF#115 construct. OsNF-YC11 or OsNF-YC12 was subcloned into modified pGADT7 without the GAL4-activation domain. LAX1-BD was used as a positive control. (F) A hypothetical model showing how OsNF-YB1 functions during grain filling. In aleurone layer cells, OsNF-YB1 is imported into nucleus through heterodimerization with two aleurone-preferentially expressed factors, OsNF-YC11 or OsNF-YC12. The heterodimer OsNF-YB1–YC11 (or YC12) interacts with OsERF#115 to form a transcriptional complex, which regulates the expression of target genes bearing the GCC box, to regulate the nutrient transportation into endosperm and further the rice filling and seed size/quality. OsERF#115 binds to the GCC box and provides the sequence specificity of the transcriptional complex.

Yeast two-hybrid assays showed that OsNF-YB1 could interact with OsERF#115, but not other ERF members (Fig. 6A). In addition, there was no interaction of OsERF#115 with OsNF-YC11 or OsNF-YC12 (Fig. 6B), indicating the specific interaction between OsNF-YB1 and OsERF#115. Although the *in vivo* interaction of OsNF-YB1 with OsERF#115 protein was hardly detected after many attempts (due to the high background), which indicates a weak interaction of OsNF-YB1 with OsERF#115 *in vivo*, identification of the GCC box by CHIP-seq analysis suggests the possible *in vivo* interaction between OsNF-YB1 and ERF proteins.

Considering that ERF transcription factor binds to the GCC box and OsERF#115 interacts with OsNF-YB1, we further performed yeast one-hybrid analysis and results confirmed that OsERF#115 binds to the GCC box, while OsNF-YB1 does not (Fig. 6C). This indicates that binding of OsNF-YB1 to the GCC box is probably mediated by OsERF#115, which is consistent with the ChIP-seq analysis revealing that OsNF-YB1 preferably binds to the GCC box and previous studies showing that NF-YB1 does not have the ability to recognize specific DNA motif.

Further transcriptional activity assay using yeast cells showed that OsEFF#115 did not possess transactivational activity (Fig. 6D), and there was no transactivational activity of OsEFF#115 in the presence of OsNF-YB1 or OsYC11 (OsYC12), or even both OsNF-YB1 and OsNF-YC11 (OsNF-YC12) (Fig. 6E).

## Discussion

### Crucial roles of OsNF-Y factor in regulating endosperm development and grain filling

Our studies have identified a possible novel non-canonical NF-Y trimeric complex consisting NF-YB, NF-YC and ERF protein that forms in rice aleurone layer cells and plays important roles in regulating endosperm development and hence grain filling. OsNF-YB1 is imported into the nucleus through heterodimerization with OsNF-YC11 or OsNF-YC12, which are preferentially expressed in aleurone layer cells. By forming the transcriptional complexes with transcription factor OsERF#115, OsNF-YB–NF-YC–ERF regulates the expression of target genes containing the GCC box and hence grain filling and endosperm development. Enrichment of transport-related genes reveals the crucial role of the OsNF-Y factor and the aleurone layer in regulating nutrient transport into endosperm, and further in rice, filling and seed size/quality (Fig. 6F).

Studies of the crystal structure of NF-Y trimer bound to DNA showed that while the NF-YB–NF-YC dimer interacts with DNA non-specifically, NF-YA provides sequence-specific contact to the CCAAT box (Nardini *et al.*, 2013). As OsNF-YB1 is capable of heterodimerizing with NF-YC but is impaired in association with NF-YA (Masiero *et al.*, 2002), the presence of another ‘DNA-reading’ subunit will provide the sequence recognition capability. Consistent with the previous studies showing that OsNF-YB1 is severely crippled in CCAAT binding capacity (Masiero *et al.*, 2002), the sequence CCAAT was not enriched in OsNF-YB1 binding peaks, whereas the GCC box was found to be enriched. Our results for the first time demonstrate that an ERF transcription factor interacts with NF-YB members to form a trimeric complex and it is proposed that the ERF transcription factor provides the sequence specificity for the GCC box.

In addition to the GCC box, the GGHGGA motif is also enriched in the binding peaks (Fig. 5D), suggesting the possible interactions between OsNF-YB1 and other transcription factors although the factors binding the GGHGGA motif have not been identified yet. It would therefore be of considerable interest to investigate whether ERF and other transcription factors compete for interactions with the NF-YB–NF-YC dimer, or whether they function together to regulate the expression of downstream targets.

Our results demonstrate the importance of *NF-Y* genes during endosperm development. Previous studies showed that several *NF-Y* family members are highly expressed in developing rice endosperm (Xue *et al.*, 2012), a number of wheat NF-Y members from each subunit family are expressed predominantly in endosperm (Stephenson *et al.*, 2007), and some *Arabidopsis NF-Y* genes are highly expressed in endosperm (by GENEVESTIGATOR database), suggesting that NF-Y complexes may play important roles in endosperm development of various plant species.

The ERF subfamily belongs to the AP2/ERF superfamily and our previous analysis (Xue *et al.*, 2012) revealed that some ERF members are predominantly expressed in endosperm. Given that OsNF-YB1 interacts with OsERF#115, it is tempting to speculate that ERF proteins may also play a role in rice endosperm development. Indeed, OsLEC1, an NF-YB subunit, interacts with APETALA2 (AP2) transcription factor SNB (Zhang and Xue, 2013), and *Arabidopsis* AP2 has a significant effect on early endosperm development (Ohto *et al.*, 2009).

OsNF-YB1 regulates grain filling and grain chalkiness, and it is interesting that enhanced *OsNF-YB1* expression results in decreased grain chalkiness, which may help to improve rice grain quality by decreasing the chalkiness.

### Functional mechanism underlying NF-Y’s function

The specific nuclear localization of OsNF-YB1 in aleurone layer cells is possibly due to the interaction with aleurone layer cell-preferentially expressed OsNF-YC11 or OsNF-YC12. In *Arabidopsis*, NF-YB proteins are detected in both the cytosol and the nucleus, and NF-YCs are detected mainly in the nucleus (Liu and Howell, 2010; Hackenberg *et al.*, 2012). It has been suggested that plant NF-YB is imported into the nucleus by interacting with NF-YC through a piggyback mechanism (Hackenberg *et al.*, 2012). OsNF-YB1 and OsNF-YCs (Supplementary Fig. S6E) show a dual cytosolic–nuclear localization in tobacco cells and are accumulated in the nucleus after coexpression, suggesting that the nuclear import of NF-YB and NF-YC requires dimer formation, which is consistent with the observations in mammals (Kahle *et al.*, 2005).

Although plant *NF-Y* genes play important roles in diverse processes, the molecular mechanisms remain unclear and genome-wide analysis of plant NF-Y binding is still deficient. The ChIP-seq analysis in this study provides informative clues to the relevant mechanisms and reveals that a number of transport-related genes are directly regulated by OsNF-YB1. In mammals, NF-Y is a pioneer factor in establishing active promoters and recent studies revealed that the role of NF-Y in transcription is to stimulate the binding of other TFs through promoting chromatin accessibility (Nardini *et al.*, 2013; Oldfield *et al.*, 2014). Considering that OsEFR#115 doesn’t possess transcriptional activity, even in the presence of OsNF-YB or OsNF-YC, although the pioneer role of plant NF-Y has not been studied in detail, it is proposed that OsNF-YB1 may promote the binding of ERF to promoter regions, and hence enhance the expression of target genes. As ERF-specific antibody is not available yet, we are not able to demonstrate reduced ERF occupancy at NF-Y-dependent sites in aleurone layer cells of *OsNF-YB1* RNAi plants.

In mammals, in addition to the core promoters, a comparable number of NF-Y binding sites are within enhancers away from transcription start sites (TSSs) (Oldfield *et al.*, 2014). Similarly, the NF-Y complex binds the *FT* distal enhancer elements in *Arabidopsis* (Cao *et al.*, 2014). It thus would be of considerable interest to know whether other plant NF-Y complexes bind enhancer elements away from TSSs, but few studies have investigated plant enhancers. Further elucidation of enhancer sites genome-wide will provide a foundation for investigating the distal NF-Y binding sites and identifying more NF-Y target genes in plants.

It has been noticed that among the 743 genes associated with OsNF-YB1 binding sites, only 10 genes are differentially expressed in *OsNF-YB1* RNAi plants. Similar poor correlations between ChIP and transcriptomic analyses were found for another plant NF-Y subunit, LEC1 (Junker *et al.*, 2012), which is reasonable for analysis at the genome-wide level. The poor correlations may be due to the involvement of other factors in OsNF-YB1 functions, or possibly, the presence of indirect regulation in which some OsNF-YB1 targets have the ability to regulate other genes (which are identified by transcriptional profile studies), resulting in decreased correlations between direct and regulated targets and a complicated transcriptional regulatory network. In addition, the RNA-seq data are from *OsNF-YB1* RNAi plants, and TFs possibly involved in OsNF-YB1 function may conceal the OsNF-YB1 target genes; the ChIP analysis using OsNF-YB1-overexpressing lines may result in binding to some genes that are not bound or regulated by OsNF-YB1 on a WT background.

A recent report showed that OsNF-YB1 regulates the expression of sucrose transporters through direct binding (Bai *et al.*, 2015), suggesting that OsNF-YB1 may regulate the target gene expression independently, in addition to the OsNF-YB–NF-YC–ERF complex. Further investigation will facilitate the understanding of the NF-Y effects and the regulatory mechanism of the NF-Y family.

### Aleurone layer is important for grain filling

There is no transfer layer in rice endosperm and the aleurone layer is proposed to be critical for apoplastic uptake of nutrients; however, little is still known about the functions and mechanisms of the aleurone layer during rice gain filling. Suppressed expression of *OsNF-YB1* results in reduced grain filling and reduced expressions of transport and ATP synthesis-related genes, demonstrating the role of OsNF-YB1 and aleurone layer in nutrient transport and endosperm development. In addition, the *OsNF-YB1* promoter, which is suitable for aleurone layer-specific expression of genes of interest in rice, provides the possibility to enhance nutrient uptake from the maternal tissues through the aleurone layer, to improve grain quality or to protect filial seeds from pathogen attack.

## Supplementary data

Supplementary data are available at *JXB* online.

Figure S1. *OsNF-YB1* is specifically expressed in aleurone layer of rice endosperm.

Figure S2. Analysis of *OsNF-YB1* RNAi transgenic plants.

Figure S3. Altered starch quality of *OsNF-YB1* RNAi transgenic plants.

Figure S4. Enhanced expression of *OsNF-YB1* in ZH11 plants transformed with OsNF-YB1 driven by native promoter (*pOsNF-YB1:OsNF-YB1*).

Figure S5. A dual cytosolic–nuclear localization of OsNF-YB1 in onion and tobacco epidermal cells.

Figure S6. Expression pattern and subcellular localization of OsNF-YCs.

Figure S7. Overview of the ChIP assays.

Table S1. List of the primers used in this study.

Table S2. List of the examined genes identified by RNA-seq or Chip-seq analysis.

Table S3. Expression of *OsERF* genes in rice aleurones.

Supplementary Data
