# Supplementary material for: Rice aleurone layer specific OsNF-YB1 regulates grain filling and endosperm development by interacting with an ERF transcription factor
Source: J Exp Bot. 2016 Nov 1;67(22):6399–411. doi: 10.1093/jxb/erw409 (PMC5181583; doi:10.1093/jxb/erw409)
Supplement: Supplementary Data [file supp_67_22_6399__index.html]

Rice aleurone layer specific OsNF-YB1 regulates grain filling and endosperm development by interacting with an ERF transcription factor — Rice aleurone layer specific OsNF-YB1 regulates grain filling and endosperm development by interacting with an ERF transcription factor — Supplementary Data 

# Rice aleurone layer specific OsNF-YB1 regulates grain filling and endosperm development by interacting with an ERF transcription factor

## Supplementary Data

Data files

- Supplementary\_Figures\_S1\_S7\_Tables\_S1\_S3.pdf - Supplementary Data
