# Supplementary material for: Rice aleurone layer specific OsNF-YB1 regulates grain filling and endosperm development by interacting with an ERF transcription factor
Source: J Exp Bot. 2016 Nov 1;67(22):6399–411. doi: 10.1093/jxb/erw409 (PMC5181583; doi:10.1093/jxb/erw409)
Supplement: Supplementary Data [file supp_erw409_Supplementary_Figures_S1_S7_Tables_S1_S3.pdf]

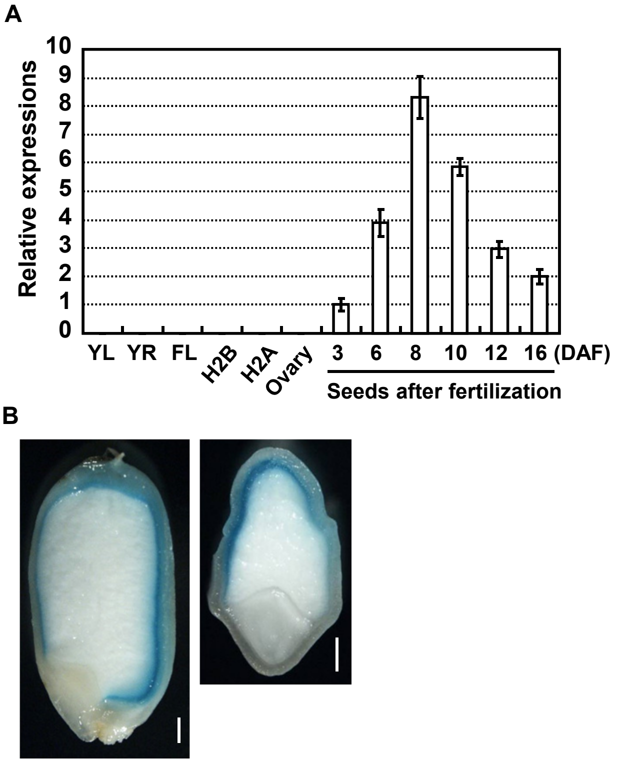

Supplementary Fig. S1. *OsNF-YB1* is specifically expressed in aleurone layer of rice endosperm.

(A) qRT-PCR analysis reveals the seed-preferential expression of *OsNF-YB1*. Various tissues including young leaf (YL) and root (YR) of seedling at three-leaf stage, flag leaf (FL) at 1 day after heading, hull at 2 days before (H2B) or after (H2A) heading, developing seeds at 3, 6, 8, 10, 12 and 16 days after fertilization (DAF), were analyzed. Data are presented as mean ± SE (n = 3).

(B) Promoter-GUS fusion analysis reveals the specific expression of *OsNF-YB1* in aleurone layer of seeds. Ten independent transgenic lines were analyzed and longitudinal section (left) and transverse section (right) of seed at 9 DAF were shown. Bars = 500 µm.

Xu et al., Supp Fig 2

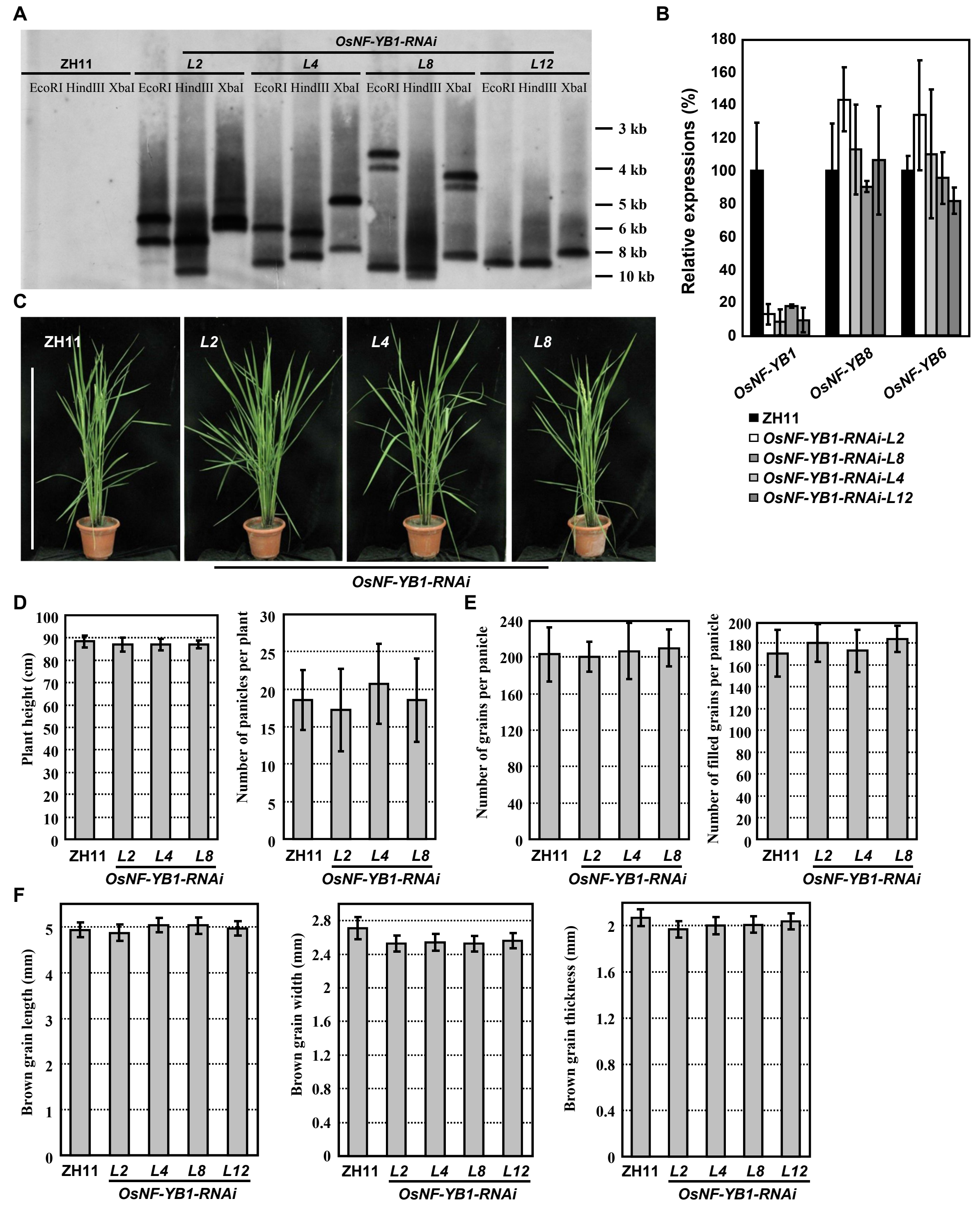

Supplementary Fig. S2. Analysis of *OsNF-YB1* RNAi transgenic plants.

(A) Southern blot analysis of *OsNF-YB1* RNAi transgenic lines. Genomic DNAs of transgenic lines and ZH11 were digested with different restriction enzymes, and blot was hybridized using digoxigenin-labeled hygromycin gene as probe.

(B) qRT-PCR analysis confirmed the suppressed expression of *OsNF-YB1* and unaltered expression of *OsNF-YB6* and *OsNF-YB8* in different *OsNF-YB1* RNAi lines. Seeds at 6 DAF were analyzed. Relative expression was calculated (expressions of corresponding genes in ZH11 was set as 100%) and data are shown as mean ± SE (n = 3).

(C) Phenotypic observation showed that *OsNF-YB1* RNAi transgenic plants do not present obvious growth changes at heading stage. Bar = 1 m.

(D-E) Unaltered height and panicle number (D) or grain number and filled grain number per panicle (E), of *OsNF-YB1* RNAi transgenic plants. Data are shown as mean ± SD (n = 10).

(F) Measurement of the length, width, and thickness of brown grains indicated the reduced grain width and thickness of *OsNF-YB1* RNAi transgenic plants. Data are shown as mean ± SD (n = 60).

Xu et al., Supp Fig 3

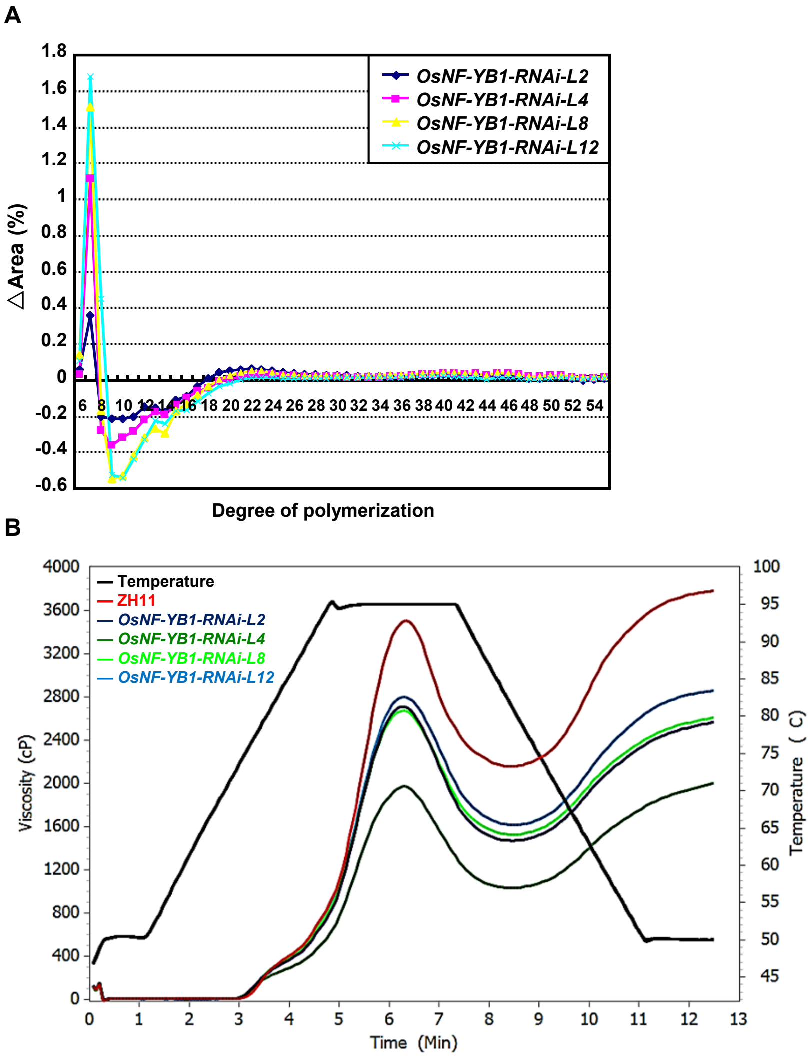

Supplementary Fig. S3. Altered starch quality of *OsNF-YB1* RNAi transgenic plants.

(A) Altered chain-length distribution of amylopectin of *OsNF-YB1* RNAi plants. Data are presented as means from three replicates.

(B) Pasting properties of endosperm starch of *OsNF-YB1* RNAi and ZH11 plants. Black line indicates the temperature changes during the measurement.

Xu et al., Supp Fig 4

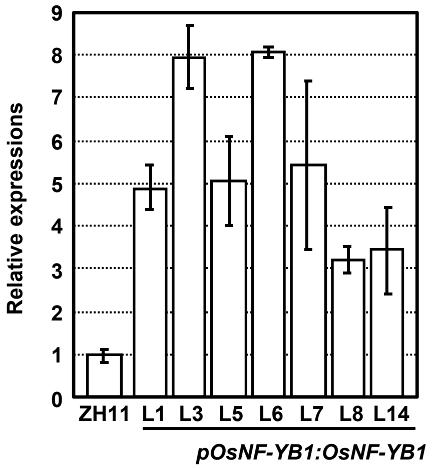

Supplementary Fig. S4. Enhanced expression of *OsNF-YB1* in ZH11 plants transformed with OsNF-YB1 driven by native promoter (*pOsNF-YB1:OsNF-YB1*). Total RNAs extracted from 6 DAF grains of 7 independent transgenic lines were analyzed by qRT-PCR and relative expressions were shown (*OsNF-YB1* expression in ZH11 was set as 1.0).

Xu et al., Supp Fig 5

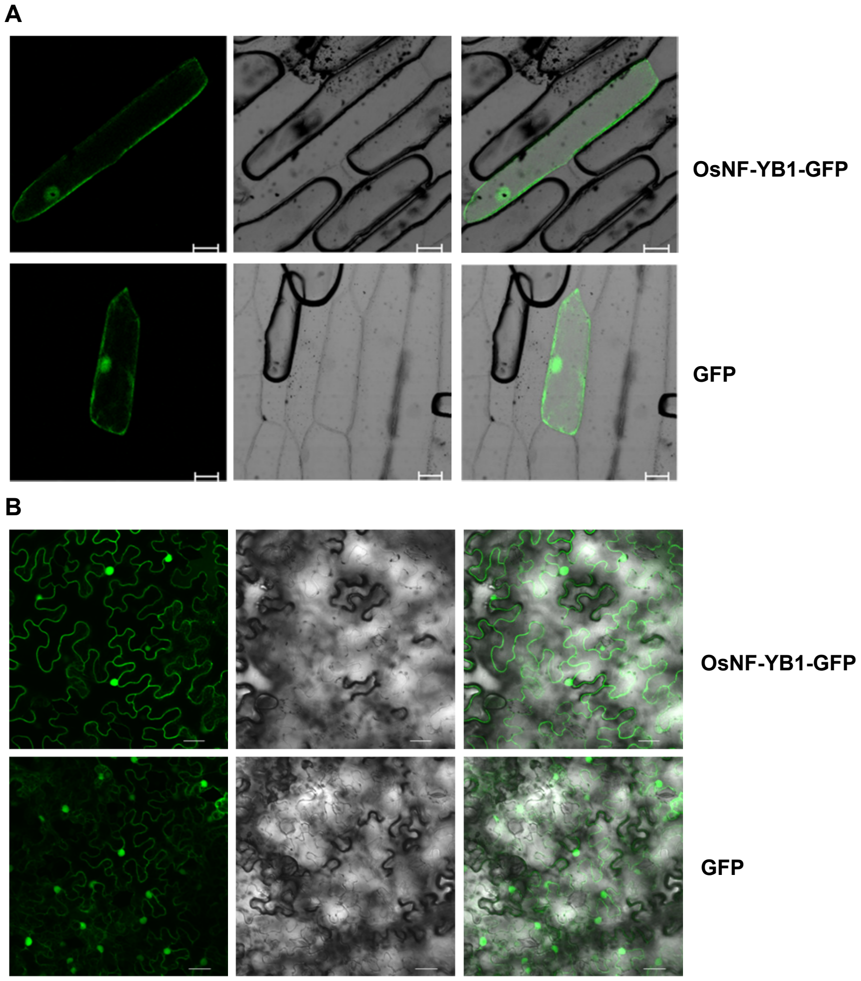

Supplementary Fig. S5. A dual cytosolic-nuclear localization of OsNF-YB1 in onion and tobacco epidermal cells. OsNF-YB1 was N-terminally fused to GFP and transiently expressed in onion (**A**, bars = 50 μm) or tobacco (**B**, bars = 20 μm) epidermal cells, and observed.

Xu et al., Supp Fig 6

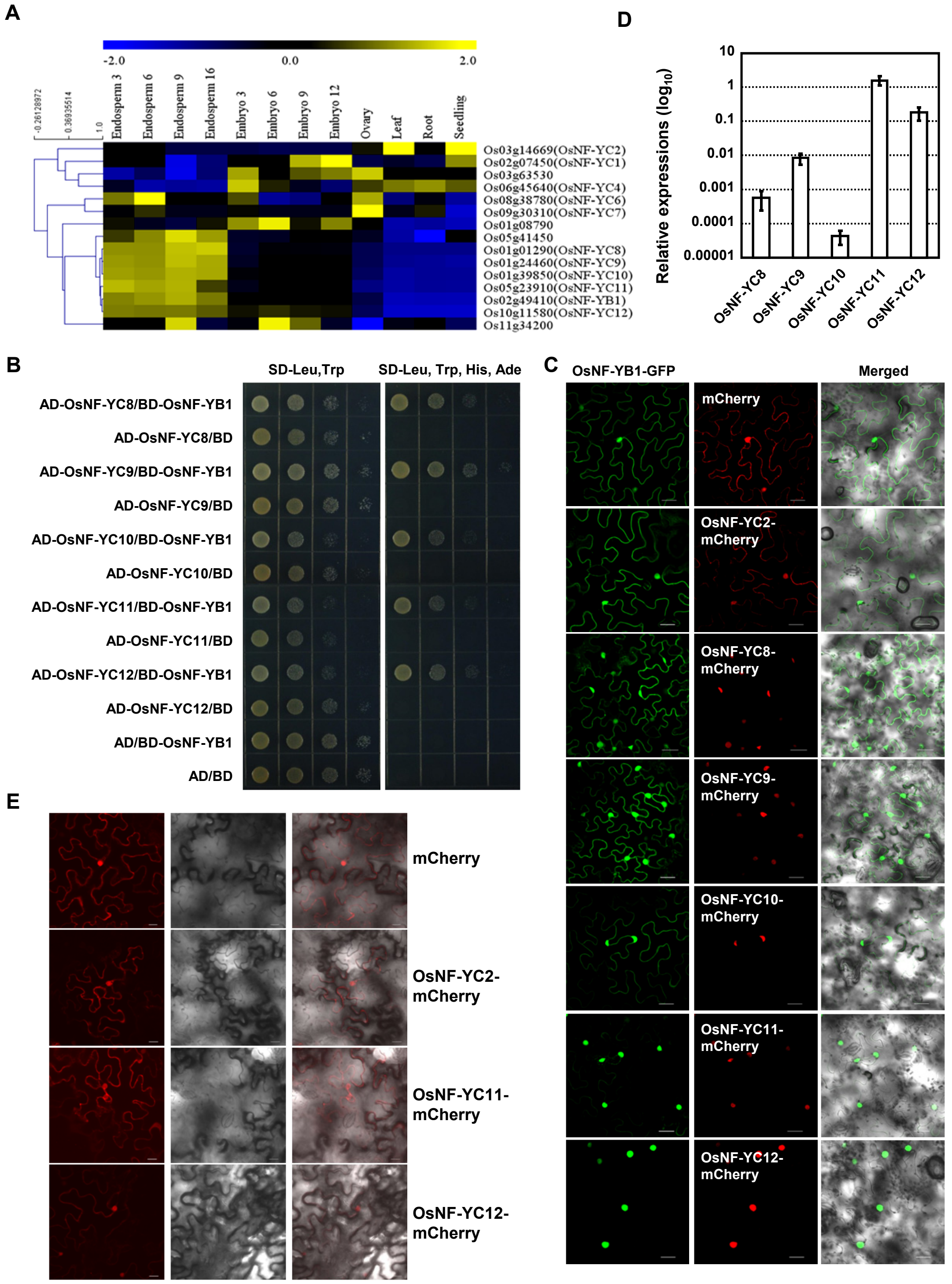

Supplementary Fig. S6. Expression pattern and subcellular localization of OsNF-YCs.

(A) Heat map showed the expression pattern of rice *OsNF-YC* genes during endosperm and embryo development and various tissues including ovary, root, leaf and seedlings. Expression pattern of *OsNF-YC* genes in ZH11 are obtained from Gene Expression Omnibus (GSE11966 and GSE27856) and bar represents the scale of relative expression levels ($\log_2$).

(B) Yeast two-hybrid assays showed the interactions between OsNF-YB1 and five OsNF-YC members. Serial dilutions (10X) of yeast cells expressing the indicated proteins were plated onto nonselective medium (SD/-Leu/-Trp) (left) or selective medium (SD/-Leu/-Trp/-Ade/-His) (right). OsNF-YC members and OsNF-YB1 were fused to activation domain (AD) or binding domain (BD) respectively.

(C) Compared to a dual cytosolic-nuclear localization in the presence of mCherry, OsNF-YC2-mCherry, OsNF-YC8-mCherry, OsNF-YC9-mCherry, or OsNF-YC10-mCherry, co-expression of OsNF-YC11-mCherry or OsNF-YC12-mCherry result in the nuclear localization of OsNF-YB1-GFP protein in tobacco cells. *OsNF-YC2* is preferentially expressed in vegetative tissues and used as a control. Bars = 30 µm.

(D) qRT-PCR analysis showed the significantly high expression of *OsNF-YC11* and *OsNF-YC12* in aleurone layer cells of 10 DAF seeds. Relative expression of *OsNF-YC* genes compared to *ACTIN* expression ($\log_{10}$) was shown (mean ± SE, $n = 3$).

(E) Observations showed that OsNF-YCs-mCherry presented a dual cytosolic-nuclear localization. OsNF-YC2, 11 or 12 were N-terminally fused to mCherry and transiently expressed in tobacco epidermal cells. Bars = 20 µm.

Xu et al., Supp Fig 7

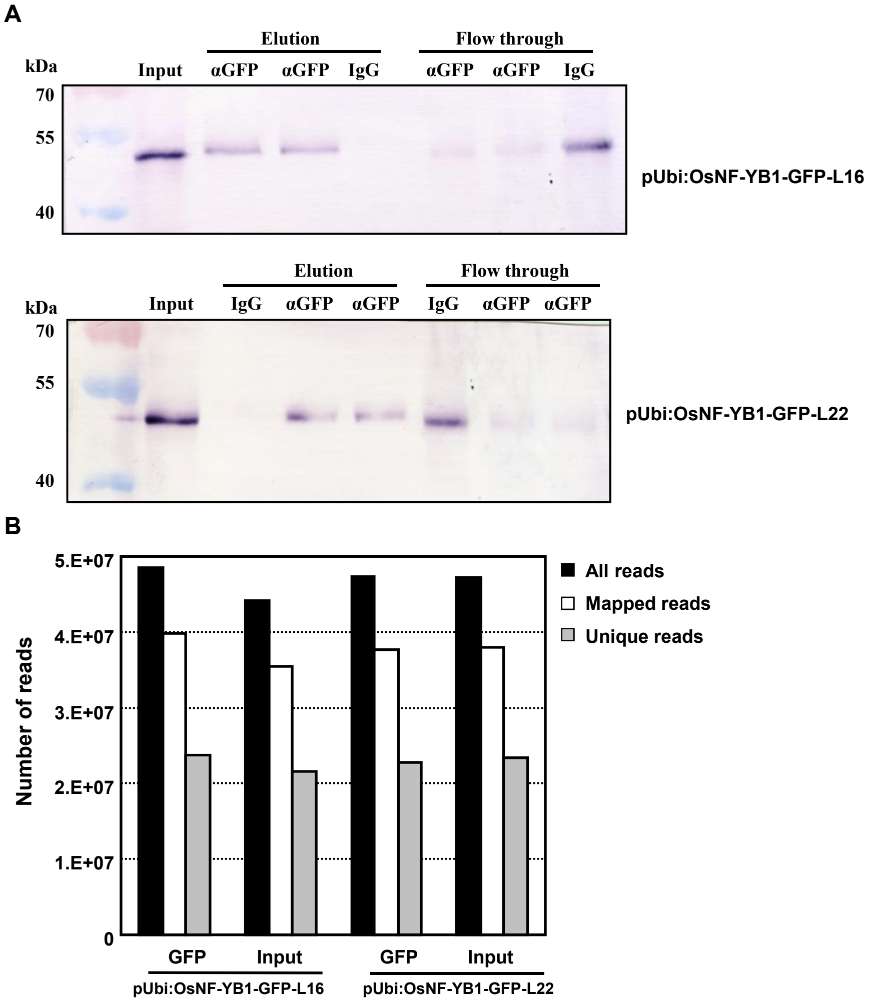

Supplementary Fig. S7. Overview of the ChIP assays.

(A) Nuclear extracts from two independent transgenic lines expressing *pUbi:OsNF-YB1-GFP* were incubated with anti-GFP antibody and normal rabbit IgG was used as a negative control. Precipitated samples (Elution) and flow-through were analyzed by Western blotting using anti-GFP antibody.

(B) Number of all reads, mapped reads and unique reads in each data set.

Supplementary Table 1. List of the primers used in this study. Added restriction enzyme sites are underlined

**Primers for vector construction**		
**Primer names**	**Sequence (5'-3')**	**Description**
RNAi-s	GGGGTACCAAGCTTAAAGCGTGGTGGCAGGAAC	RNAi construct of OsNF-YB1
RNAi-a	CGGGATCCCTGCAGTGGGGTCGACGTAGCGATC	
GUS-s	GCTCTAGATAAGAAATCGCTCTTCCTC	OsNF-YB1 promoter-GUS fusion
GUS-a	CGGGATCCGCTCTCTCAAGTCTCAATGA	
In situ-s	ACGCCCGGTGGACCGAC	*In situ* hybridization analysis
In situ-a	ACGCAAACATCAAGCATT	
GFP-s	CCCTCGAGATGGCAGGGAACAAAAAG	OsNF-YB1-GFP fusion
GFP-a	GACTAGTTGCATATTTTTTCCATAGCCAT	
OsNF-YC2-mCherry-s	CGGGATCCATGGACAACCAGCAGCTA	OsNF-YC2-mCherry fusion
OsNF-YC2-mCherry-a	GGACTAGTTTCGGAGCTTGGAGGTGC	
OsNF-YC8-mCherry-s	CGGGATCCATGGAGCAAACTTTGGAC	OsNF-YC8-mCherry fusion
OsNF-YC8-mCherry-a	CGCGTCGACGGAATGGTCTTCATGCAA	
OsNF-YC9-mCherry-s	CGGGATCCATGAAGCAAACTTTGGAT	OsNF-YC9-mCherry fusion
OsNF-YC9-mCherry-a	CGCGTCGACCTTGTTGCCATTACTGGT	
OsNF-YC10-mCherry-s	CGGGATCCATGGAGCAAACTTTGGAC	OsNF-YC10-mCherry fusion
OsNF-YC10-mCherry-a	CGCGTCGACCTTATTGGCATTGCTTGT	
OsNF-YC11-mCherry-s	CGGGATCCATGGCGATTCCAACAAAG	OsNF-YC11-mCherry fusion
OsNF-YC11-mCherry-a	CGCGTCGACTTTCTCATGAAGAAATAG	
OsNF-YC12-mCherry-s	CGGGATCCATGCCGATCCCGGAAAAG	OsNF-YC12-mCherry fusion
OsNF-YC12-mCherry-a	CGCGTCGACCTCAGGAAGATCTTTGTC	
OsNF-YC2-RFP-s	CGGGGTACCATGGACAACCAGCAGCTA	OsNF-YC2-RFP fusion
OsNF-YC2-RFP-a	GCTCTAGACTATTCGGAGCTTGGAGG	
OsNF-YC11-RFP-s	CGGGGTACCATGGCGATTCCAACAAAG	OsNF-YC11-RFP fusion
OsNF-YC11-RFP-a	GCTCTAGATTATTTCTCATGAAGAAA	
OsNF-YC12-RFP-s	CGGGGTACCATGCCGATCCCGGAAAAG	OsNF-YC12-RFP fusion
OsNF-YC12-RFP-a	GCTCTAGATCACTCAGGAAGATCTTT	
OsNF-YB1-BD-s	GGAATTCATGGCAGGGAACAAAAAG	pGBKT7-OsNF-YB1
OsNF-YB1-BD-a	CGGGATCCCCATATTTTTTCCATAGCCAT	
OsNF-YC8-AD-s	CGCCATATGATGGAGCAAACTTTGGAC	pGADT7-OsNF-YC8
OsNF-YC8-AD-a	CGGAATTCCTAGGAATGGTCTTCATG	
OsNF-YC9-AD-s	CGCCATATGATGAAGCAAACTTTGGAT	pGADT7-OsNF-YC9
OsNF-YC9-AD-a	CGGAATTCTTACTTGTTGCCATTACT	
OsNF-YC10-AD-s	CGCCATATGATGGAGCAAACTTTGGAC	pGADT7-OsNF-YC10
OsNF-YC10-AD-a	CGGAATTCTTACTTATTGGCATTGCT	
OsNF-YC11-AD-s	CGCCATATGATGGCGATTCCAACAAAG	pGADT7-OsNF-YC11
OsNF-YC11-AD-a	CGGGATCCTTATTTCTCATGAAGAAA	
OsNF-YC12-AD-s	CGCCATATGATGCCGATCCCGGAAAAG	pGADT7-OsNF-YC12
OsNF-YC12-AD-a	CGGAATTCTCACTCAGGAAGATCTTT	
OsERF#074-AD-s	CGCCATATGATGGCGCCGAGAACGTCG	pGADT7-OsERF#074
OsERF#074-AD-a	CGGAATTCCTAGACCTCCATCGGCGG	
OsERF#114-AD-s	CGCCATATGATGCCGCCGCCTGCAGCC	pGADT7-OsERF#114
OsERF#114-AD-a	CGGAATTCTCATTCGAAATTGGATGC	
OsERF#115-AD-s	CGCCATATGATGGTGCCGCCGGCGGCG	pGADT7-OsERF#115
OsERF#115-AD-a	CGGAATTCTCACTCTCCTTGCTGAAC	
OsERF#072-AD-s	CGGAATTCATGTGCGGCGGAGCAATC	pGADT7-OsERF#072
OsERF#072-AD-a	CGGAATTCTCAGTAGGCACCAGCTGC	
GCC-box-pHIS2-s	AATTCCCTATGGCCGCCGCTCCTATGGCCGCCGC	pHIS2-GCC-box

	TCCTATGGCCGCCGCTGAGCT	
GCC-box-pHIS2-a	CAGCGGCGGCCATAGGAGCGGCGGCCATAGGA GCGGCGGCCATAGGG	
mGCC-box-pHIS2-s	AATTCCCTATGTCCTCCTCTCCTATGTCCTCCTCT CCTATGTCCTCCTCTGAGCT	pHIS2-mGCC-box
mGCC-box-pHIS2-a	CAGAGGAGGACATAGGAGAGGAGGACATAGGA GAGGAGGACATAGGG	

Primers for qRT-PCR analyses

Gene Name	Sense (5'-3')	Antisense (5'-3')
Actin	CCTTCAACACCCCTGCTATG	TGAGTAACCACGCTCCGTCA
OsNF-YB1	GAATATAGCGGCTCATCACC	CACACACACATGCATCAAGTT
OsNF-YB6	ACCCAACCTTTCCTCCTAAT	ACCTAGCTTCTCACCATCAT
OsNF-YB8	ACTAGCGTACGTCTCATCTC	ACATCCGACTCCTATATGACC
OsNF-YC8	GCTAATAGGTAGCATTTTCT	CAAAATTCATCCATTTGTTG
OsNF-YC9	GTTCCAATAATAGTAGCGTA	ACTATGTAAGAACGTCGAG
OsNF-YC10	ACATAGTAACAATGACACAA	GACCATTGATAACTATAGCA
OsNF-YC11	CCATAAATTGGTCACCTG	CTTGAAAATGTATTGTCCTC
OsNF-YC12	ATCAATCATCCTATAGTTGC	ATGTATCATCCTCTTGATTG
Os01g08350	TGAGAATGAGAATGTAGGTA	TTTCCATCAATAGGTACTCC
Os01g58010	TGGAGTTGTTACGAAAAAG	GTAGAAACATGAGCTTGTAA
Os12g14930	CTCTCGACGGGAGAATTGAG	TGCACTGTCTTTTCGCACTT
Os12g34018	AGACCGTCGACTATTTGGGA	AGATTATGCTTCCTTGCCCG
Os12g34108	GAAAGAAAAGCGTGACGAGC	GGACGTTTCCAATACCGACA
Os01g41720	TTGAAGCGATAAAGAAAGAC	CATCAACATTGATTCCGAAC
Os11g47520	TAATGGACGAATAAAATGAGG	TCCATACATAACCTTCTTTC
Os02g48790	AGCGAGATAGCTGGAAAACG	TTCTGGGTTCATCCTGGTCT
Os06g40704	AGTAATCAAAAGCAATCTGA	CGTCATGATCATACACTTG
Os03g08460	GCTTTAATTTTGTGAGGAAC	CCTTAATTTGCTCATGTTTG
Os07g08420	ATCACTGAGTATAGTTGGTT	TGAGCATCTGTATTTTGATT

Primers for ChIP-qPCR analyses

Gene Name	Sense (5'-3')	Antisense (5'-3')
Os01g63680	TACAAGAGAGGCAAAGAAAT	CTCTCCCATCACCTTTCC
Os02g32660	GTTCATTGACTTAGTTCGC	CTTAGCTTCCTCATCCAC
Os02g36414	AGTAAGTCTTGCGTTCTAAT	GAGGAAGAAGTGGAAGATG
Os03g43720	CTCTCTTGCAGCTTCATT	GAACACGAGAAACTCGAT
Os04g33720	CTTGCCGATCACCGATA	GTGATGGTGCTGAGGAG
Os05g32900	CAACTACTGCAAGTTTGA	ATACCGAAGACAATCATGG
Os06g03970	ATGCACTAGTGACACAAC	TAGCTTATTCCCTCTCAAG
Os07g19070	TAAGAGTTGAAGAAGGTAGG	TAGCAATGGCTGTCGTAG
Os10g17890	CCATTGGCATGGATTTGG	CTCATGTCCACACCCAAC
Os10g36110	CACATCAACCAGTAATCAAT	CCACTTCGCCATTGCTC

Supplementary Table 2. List of the examined genes identified by RNA-seq or ChIP-seq analysis.

Locus	Gene functions
Os01g08350	ATP synthase
Os01g58010	ATP synthase
Os12g14930	ATP synthase
Os12g34018	ATP synthase
Os12g34108	ATP synthase
Os01g41720	Metal ion transport, transmembrane transport
Os11g47520	Glycosyl hydrolase, putative
Os02g48790	AML1, putative
Os06g40704	Stromal membrane-associated protein, putative
Os03g08460	OsEBP-89
Os07g08420	RISBZ1
Os01g63680	MYB family transcription factor
Os02g32660	OsBEIIb
Os02g36414	Sugar transporter
Os03g43720	Sugar transporter
Os04g33720	OsCIN3, cell wall invertase
Os05g32900	Hexose carrier protein HEX6
Os06g03970	OsSIK1, receptor-like kinase
Os07g19070	Prefoldin
Os10g17890	OsWAK113
Os10g36110	LTPL158

Supplementary Table 3. Expression of *OsERF* genes in rice aleurones. RNAs extracted from aleurones (8 days after fertilization) were used for RNA-seq analysis and FPKM (Reads Per Kilobase of exon model per Million mapped reads) value of *OsERF* genes were shown (those of *OsERF#115*, *OsERF#072* and *OsERF#074* are highlighted).

Gene	Locus	FPKM
OsERF#115	Os08g41030	4234.92
OsERF#129	Os04g56150	459.968
OsERF#072	Os09g26420	353.711
OsERF#061	Os05g29810	249.76
OsERF#074	Os05g41780	160.602
OsERF#108	Os01g04020	140.674
OsERF#068	Os01g21120	128.739
OsERF#070	Os02g54160	99.4018
OsERF#075	Os01g58420	70.1135
OsERF#064	Os03g08500	57.736
OsERF#058	Os03g60120	36.6665
OsERF#076	Os04g57340	34.3101
OsERF#077	Os04g52090	32.9877
OsERF#057	Os07g12510	28.7904
OsERF#071	Os06g09390	24.1007
OsERF#103	Os02g52670	18.885
OsERF#067	Os07g47790	18.2951
OsERF#062	Os03g08470	16.399
OsERF#134	Os02g09650	13.9602
OsERF#102	Os09g28440	13.049
OsERF#099	Os01g64790	12.5725
OsERF#113	Os06g42990	11.2212
OsERF#056	Os05g25260	10.0316
OsERF#060	Os03g08460	9.77545
OsERF#114	Os06g42910	8.45776
OsERF#121	Os06g47590	7.36757
OsERF#095	Os02g43820	5.60944
OsERF#053	Os01g12440	5.06431
OsERF#105	Os05g36100	4.68561
OsERF#130	Os05g41760	3.78437
OsERF#104	Os08g36920	2.73722
OsERF#109	Os09g13940	2.33968
OsERF#132	Os02g06330	1.53752
OsERF#054	Os01g46870	1.31582
OsERF#125	Os02g34270	1.22472
OsERF#106	Os08g42550	1.16661
OsERF#093	Os04g46220	1.10002
